# Retrospective Evaluation of Deep Transcranial Magnetic Stimulation as Add-On Treatment for Parkinson’s Disease

**DOI:** 10.3389/fneur.2015.00210

**Published:** 2015-10-26

**Authors:** Francisco Torres, Esteban Villalon, Patricio Poblete, Rodrigo Moraga-Amaro, Sergio Linsambarth, Raúl Riquelme, Abraham Zangen, Jimmy Stehberg

**Affiliations:** ^1^Neuromagnetics, Santiago, Chile; ^2^Laboratorio de Neurobiología, Centro de Investigaciones Biomédicas, Universidad Andres Bello, Santiago, Chile; ^3^Neuroscience Laboratory, Ben-Gurion University of the Negev, Beersheva, Israel

**Keywords:** Parkinson’s disease, repetitive transcranial magnetic stimulation, H-coil, motor cortex, prefrontal cortex, high and low frequency, deep TMS

## Abstract

**Objective:**

To evaluate the safety and assess the different symptom improvements found after a combined low-frequency primary motor cortex and high-frequency prefrontal cortex (PFC) stimulation using the deep TMS (dTMS) H-coil, as an add-on treatment for Parkinson’s disease (PD).

**Methods:**

Forty-five PD patients underwent 14 dTMS sessions; each consisting of 1 Hz stimulation of the primary motor cortex for 15 min, followed by 10 Hz stimulation of the PFC for 15 min. Clinical assessments were performed, BEFORE, at the MIDDLE, and END of therapy as well as at FOLLOW-UP after 30 days, using Movement Disorder Society-Unified Parkinson’s Disease Rating Scale, TINETTI, UP&GO, SCOPA, HDRS_21_, Beck Depression Inventory, and self-applied daily motor assessment scales.

**Results:**

Treatment was well-tolerated, without serious adverse effects. dTMS-induced significant PD symptom improvements at END and at FOLLOW-UP, in all subscales of the UPDRS, gait speed, depressive symptoms, balance, autonomic symptoms, and a 73% increase in daily ON time.

**Conclusion:**

In the cohort of PD patients treated, dTMS was well-tolerated with only minor adverse effects. The dTMS-induced significant improvements in motor, postural, and motivational symptoms of PD patients and may potentiate concurrent levodopa treatment.

**Significance:**

The present study demonstrates that dTMS may have a much wider spectrum of beneficial effects than previously reported for TMS, including enhancement of levodopa effects, suggesting that future clinical trials with dTMS should include a broader range of symptom measurements.

## Introduction

Parkinson’s disease (PD) is a chronic, progressive disorder for which there is no satisfactory long-term treatment. The gold standard pharmacological treatment for PD is orally administered levodopa. Levodopa, while highly effective at controlling motor symptoms for a limited time, shows progressive decrease in efficacy as the disease progresses. This demands a constant increase in dosage to manage symptoms, resulting in a mirrored increase in treatment-related adverse effects. This has led to the development of several drugs, including dopaminergic agonists, which are combined with levodopa to improve PD symptoms and control its adverse effects. As the disease progresses, PD symptoms and adverse effects re-emerge, highlighting the need for the development of novel therapies that can improve PD symptoms and related comorbidities.

Focal neuromodulation with repetitive transcranial magnetic stimulation (rTMS) has raised increased interest over the past few years as a promising coadjuvant treatment for many neurological and psychiatric disorders, including PD, as it induces changes in cortical excitability non-invasively (1, 2) that persist after stimulation, with cumulative effects over time (3).

Several studies have found that high-frequency stimulation of the motor cortex with rTMS (5–10 Hz) improves motor symptoms in PD patients (1, 4–9) and produces enduring cortical excitation (2, 10–12).

However, several studies have reported that PD patients suffer from impaired intra-cortical inhibition (13–17), suggesting that low-frequency stimulation of the motor cortex may be more beneficial in PD. Indeed, rTMS of the primary motor cortex (M1) with low frequencies (0.1–1 Hz) has been shown to produce significant improvement in motor symptoms of PD patients (6, 18) and long-lasting increases in intra-cortical inhibition (19–21). A recent meta-analysis that included eight randomized placebo-controlled trials that used low frequency rTMS to M1 showed significant improvements in PD motor symptoms compared to placebo (22).

High-frequency rTMS of the prefrontal cortex (PFC) has also been shown to improve motor symptoms (23). This improvement is potentially explained by an increase in striatal dopamine release (24).

A new coil for TMS, known as deep TMS H-coil (dTMS) designed to stimulate deep cortical areas with lower intensities than conventional coils (25) has been reported to induce significant improvements in motor symptoms (26) in PD patients. The objective of the present study is to retrospectively evaluate the effectiveness and safety of dTMS as an add-on treatment for PD, using a combination of low frequency (1 Hz) stimulation of M1 and high-frequency stimulation of PFC, and to analyze the effects of treatment using a wide range of motor and non-motor evaluations.

## Materials and Methods

### Patients

The present study was performed according to the Declaration of Helsinki and following a protocol approved by the Ethical Committee of the Universidad Andrés Bello (0072015). Forty-five PD patients (26 men, 19 women) were included in the study and attended Neuromagnetics for dTMS treatment between October 2012 and September 2014. Each participant signed an informed consent. Patients were non-demented adults suffering from idiopathic PD, according to previously established clinical criteria (27).

The inclusion criteria consisted of a PD diagnosis, pharmacological treatment with levodopa or dopaminergic agonists, and the ability to provide oral or written informed consent. The exclusion criteria included neurological or psychiatric disorders other than PD and depression, recent head trauma, personal or family history of seizures, presence of metal implants, pacemakers or DBS, and uncompensated or non-medicated chronic medical conditions (such as hypertension or diabetes).

### Deep transcranial magnetic stimulation

A dTMS H2 *par* coil (Brainsway Inc., Israel), designed to bilaterally stimulate the complete cortical thickness (25), was used with a Rapid2 MagStim stimulator (MagStim Company, Ltd., Carmarthenshire, Wales, UK). The total duration of the stimulation protocol was 3 weeks. Patients underwent 5 sessions of dTMS per week for a total of 12–16 sessions (14 sessions on average), and were evaluated at 30 days post-treatment. Each session consisted of 16 min of 1 Hz dTMS to M1 and 16 min of 10 Hz stimulation to the lateral PFC. For stimulation, the coil was first located over the M1 and tilted in the coronal/sagittal planes to a position that induced hand movements when stimulated. Resting motor threshold (RMT) was determined as the lowest stimulation intensity capable of inducing thumb movement as measured by evoked potentials using an EMG of the abductor pollicis brevis muscle. Stimulation of M1 was bilateral with stimulus intensity set at 110% of RMT (900 stimuli, 90 trains of 10 pulses at 1 Hz, 1 s inter-train interval). For PFC stimulation, the coil was moved 6 cm anterior symmetrically from the RMT location and stimulation intensity was set at 100% RMT (1000 stimuli, 50 trains of 20 pulses at 10 Hz, 18 s inter-train interval). 1 Hz stimulation of M1 always preceded 10 Hz stimulation of PFC.

### Clinical measures

Clinical assessments were performed during patients’ ON state, at the same time of day, at baseline (BEFORE), after 6 daily sessions (MID), after completion of treatment (END), and 30 days after the end of treatment (FOLLOW-UP).

All patients were evaluated with (1) Hoehn and Yahr (H&Y) stages evaluation for PD severity (28) and with (2) the Movement Disorder Society-Unified Parkinson’s Disease Rating Scale (MDS-UPDRS), including Part I [non-motor activities of daily living (ADL)], Part II (motor ADL), Part III (motor examination), and Part IV (motor complications) (29). (3) The SCOPA-AUT (30, 31) to measure autonomic symptoms in PD, including subscales for cardiovascular, urinary, sexual, thermoregulation, and gastrointestinal dysfunction, (4) the UP&GO to measure gait speed (32), (5) the Tinetti for risk of fall (33), (6) the Hamilton Rating Scale for Depression (HDRS_21_) (34), (7) the Beck Depression Inventory (BDI) (35) to assess depressive symptoms, and (8) a self-assessment of ON&OFF daily periods.

### Safety

Patients were asked to report every potential adverse effect, including side effects previously associated to TMS or dTMS, such as discomfort, headache, toothache, facial ache, seizures, pain, cognitive effects, nausea, motor effects/weakness, sleep/tiredness, and auditory effects (36, 37).

### Statistical analysis

Statistical analysis was performed using GraphPad Prism v5. All patient data were analyzed using a one-way repeated measures ANOVA to assess differences between three time-points of testing (BEFORE, MID, and END) using TIME as a factor at three levels for each scale, except for SCOPA. SCOPA was only measured at BEFORE and END and was analyzed using a paired *t*-test.

Data from the 22 patients that attended the 30-day FOLLOW-UP session were used to analyze the post-treatment effects of dTMS stimulation with the MDS-UPDRS scale. For other scales, data are available from only 16 patients that attended the FOLLOW-UP session. A one-way repeated measures ANOVA was used to assess the difference between the three time points (BEFORE, END, and FOLLOW-UP). The ON&OFF evaluation was not measured at FOLLOW-UP.

To test for differences in treatment effects between patients who were depressive and those who were not depressive BEFORE treatment, patients were divided into two groups; those that had seven or less points and those that had more than seven points in the HDRS_21_. A one-way repeated measures ANOVA was used to assess the difference between the three time points (BEFORE, END, and FOLLOW-UP) for each curve. Then, a two-way ANOVA with a Bonferroni *post hoc* test was used to assess differences between the two curves at each time point. Finally, to assess differences in the improvements in motor UPDRS for between groups, the difference between BEFORE and END for each group was compared between the groups using a paired *t*-test.

To test for differences between gender, physical activity and UP&GO improvement, ANOVA was performed with TIME (4 levels) and GENDER or SPORT or UP&GO time (2 levels). ANOVA was also performed using UP&GO initial severity (time) (1 level) and % of improvement in the UP&GO (difference in time to perform the task between BEFORE and END). *Post hoc* comparisons were performed using the Bonferroni test. Multivariate analysis and possible correlations between initial symptoms, motor improvements, and clinical demographics [age, disease duration, H&Y scale, levodopa equivalent daily dose (LEDD) and baseline motor scores] were analyzed using Spearman’s test. A *p*-value <0.05 was considered statistically significant.

## Results

Forty-five patients with PD were evaluated and treated with at least 12 sessions of dTMS, with an average of 13.6 ± 0.5 sessions. The patients were on average 62.5 ± 1.6 years old, had an H&Y score of 2.3 ± 0.2 and 9.8 ± 0.9 years since diagnosed. To see details of patient demographics see Table 1. Although 32 patients returned for assessment 30 days after treatment (FOLLOW-UP) and had their MDS-UPDRS evaluation, only 16 of them completed the evaluation for the other scales. Most patients who failed to attend the FOLLOW-UP session reported by phone not to have attended because they felt well and preferred to postpone treatment sessions while being asymptomatic. Others reported being busy and not having sufficient time to attend. From the 32 patients who attended FOLLOW-UP, 16 were only evaluated using the MDS-UPDRS reportedly because they did not have sufficient time that day to complete the other evaluations besides the MDS-UPDRS. Average LEDD was 470.72 mg at BEFORE and did not change significantly throughout the study, being 478.62 mg at END. The medications used were Levodopa (565.2 ± 9.9 mg; 80% patients); pramipexole (2.2 ± 0.1 mg; 68% of patients); hydrochlorothiazide (50 ± 0 mg; 3%); citalopram (20 ± 0 mg; 8%); Resagiline (1 ± 0 mg; 3%). Three patients were not taking medication during treatment.

**Table 1:** **Demographics and clinical characteristics of PD patients**.

	All patients			Physical activity			No physical activity		

	All	Female	Male	All	Female	Male	All	Female	Male
Number of patients	45	19	26	26	12	14	19	7	12
Average age (years)	62.5 ± 1.6	63.7 ± 2.4	61.5 ± 2.1	61.7 ± 2.0	64.4 ± 3.0	59.3 ± 2.6	63.6 ± 2.6	62.6 ± 4.3	64.2 ± 3.4
Average years disease	9.8 ± 0.9	10.4 ± 1.4	9.4 ± 1.1	10.3 ± 1.2	10.9 ± 2.0	9.9 ± 1.6	9.1 ± 1.3	9.4 ± 1.9	8.0 ± 2.4
Average severity	2.6 ± 0.1	2.7 ± 0.2	2.6 ± 0.2	2.4 ± 0.2	2.6 ± 0.2	2.5 ± 0.2	2.7 ± 0.2	2.9 ± 0.5	2.3 ± 0.4
Sessions	13.6 ± 0.5	14.2 ± 0.9	13.1 ± 0.4	13.8 ± 0.8	14.3 ± 1.5	13.3 ± 0.7	13.4 ± 0.4	14.1 ± 0.6	12.5 ± 0.5

*The number of patients, their gender, age, years of disease, disease severity (according to Hoehn and Yahr), and number of stimulation sessions are shown for all patients and subdivided according to their physical activity (all data shown as average ± SE)*.

For a detailed summary of results from all 45 patients, see Table 2 and for those patients that attended the FOLLOW-UP session see Table 3.

**Table 2:** **Summary of results**.

	*N*	Start	Middle	End	*p*-Value	**Δ**
**MDS-UPDRS**						
Non-motor aspects of activities of daily living	45	11.3 ± 0.9	8.5 ± 0.8	7.1 ± 0.8	<0.0001	4.1 ± 0.5
Motor aspects of activities of daily living	45	16.4 ± 1.1	13.8 ± 1.1	12.2 ± 1.1	<0.0001	4.3 ± 0.5
Motor examination	45	37.0 ± 2.3	34.0 ± 2.5	28.5 ± 2.3	<0.0001	8.7 ± 1.0
Motor complications	45	5.3 ± 0.7	4.3 ± 0.5	4.2 ± 0.5	<0.0001	1.4 ± 0.4
Total	45	70.0 ± 3.8	60.6 ± 4.0	50.8 ± 4.0	<0.0001	20.5 ± 2.4
**SCOPA-AUT**						
Gastrointestinal	45	3.2 ± 0.4	–	2.0 ± 0.3	<0.001	1.2 ± 0.3
Urinary	45	3.2 ± 0.5	–	1.7 ± 0.3	<0.0001	1.5 ± 0.3
Cardiovascular	45	3.6 ± 0.5	–	1.7 ± 0.3	<0.0001	2.0 ± 0.4
Thermoregulation	45	2.1 ± 0.4	–	1.6 ± 0.4	>0.05	0.7 ± 0.3
Sexual	45	1.3 ± 0.5	–	1.0 ± 0.4	>0.05	0.1 ± 0.1
Total	45	12.7 ± 1.2	–	7.5 ± 1.0	<0.0001	5.1 ± 0.7
**On-Off**						
ON time (h/day)	14	4.4 ± 1.2	7.2 ± 1.2	7.6 ± 1.2	<0.05	3.2 ± 1.5
OFF time (h/day)	14	10.4 ± 1.3	7.7 ± 1.2	7.2 ± 1.3	<0.05	3.2 ± 1.7
%ON	14	30.4 ± 8.5	48.7 ± 7.5	51.61 ± 8.2	<0.05	21.0 ± 11.1
%OFF	14	69.3 ± 8.6	51.3 ± 7.6	48.3 ± 8.2	<0.05	20.8 ± 11.1
UP&GO	45	17.0 ± 3.7	11.2 ± 0.8	9.7 ± 0.4	<0.05	7.7 ± 3.9
**TinettI**						
Gait	45	7.6 ± 0.3	9.5 ± 0.2	10.0 ± 0.2	<0.0001	2.3 ± 0.3
Static balance	45	12.0 ± 0.3	14.1 ± 0.3	14.5 ± 0.3	<0.0001	2.6 ± 0.4
BDI	45	10.7 ± 1.2	6.8 ± 0.9	5.4 ± 0.9	<0.0001	5.4 ± 1.0
HDRS-21	45	11.3 ± 1.1	6.5 ± 0.9	5.5 ± 0.9	<0.0001	5.8 ± 1.0
**RS**						
Depressive	25	19.1 ± 1.6	16.2 ± 1.7	14.8 ± 1.7	<0.0001	4.0 ± 1.1
Non-depressive	20	13.1 ± 1.1	10.8 ± 1.0	9.0 ± 0.8	<0.0001	3.1 ± 0.3

Results from the different scales are shown for each time point (Before, Middle and End) for the 45 patients. Significance is shown as *p < 0.05; **p < 0.01; ***p < 0.001; *p*-value and the difference between Before and End time points are also shown. All results appear as average ± SEM.

**Table 3:** **Summary of results from patients with 30-day follow-up**.

	*N*	Start	End	Follow-up	*p*-Value	**Δ**
**MDS-UPDRS**						
Non-motor aspects of activities of daily living	32	11.6 ± 0.9	7.3 ± 0.8	2.9 ± 0.9	<0.0001	8.7 ± 0.8
Motor aspects of activities of daily living	32	15.6 ± 1.2	10.9 ± 0.9	10.8 ± 0.9	<0.0001	4.7 ± 0.6
Motor examination	32	33.1 ± 2.6	24.8 ± 2.2	21.0 ± 2.2	<0.0001	12.1 ± 0.9
Motor complications	32	5.8 ± 0.7	4.1 ± 0.5	4.5 ± 0.6	<0.01	1.3 ± 0.7
Total	32	63.0 ± 4.3	47.1 ± 3.6	39.2 ± 3.6	<0.0001	24.3 ± 1.9
**SCOPA-AUT**						
Gastrointestinal	16	3.3 ± 0.7	2.1 ± 0.6	2.6 ± 0.7	<0.05	0.6 ± 0.5
Urinary	16	2.8 ± 0.7	1.4 ± 0.6	1.6 ± 0.7	<0.05	1.1 ± 0.5
Cardiovascular	16	3.6 ± 0.9	1.4 ± 0.5	1.3 ± 0.3	<0.01	2.2 ± 0.7
Thermoregulation	16	1.6 ± 0.5	1.2 ± 0.4	1.0 ± 0.4	>0.05	0.6 ± 0.5
Sexual	9	1.3 ± 0.7	0.9 ± 0.5	0.9 ± 0.4	>0.05	0.4 ± 0.4
Total	16	11.9 ± 1.7	6.6 ± 1.7	7.1 ± 1.5	<0.0001	4.9 ± 1.3
**TINETTI**						
Gait	16	7.3 ± 0.5	10.1 ± 0.3	9.9 ± 0.4	<0.0001	2.4 ± 0.6
Static balance	16	12.0 ± 0.7	14.7 ± 0.4	14.6 ± 0.4	<0.0001	3.2 ± 0.7
BDI	16	9.1 ± 1.6	4.9 ± 1.5	6.3 ± 1.5	<0.01	3.9 ± 0.8
HDRS-21	16	9.3 ± 1.4	3.6 ± 1.5	5.6 ± 0.7	<0.001	4.3 ± 0.6

Results from the different scales are shown for each time point (Before, End and FOLLOW-UP) for the patients who attended the FOLLOW-UP session 30 days after treatment (*N* is number of patients with data and delta corresponds to the change in scale in points). Significance is shown as *p < 0.05; **p < 0.01; ***p < 0.001; *p*-value and the difference between BEFORE and END time points are also shown. All results appear as average ± SEM.

### Safety

Treatment was well-tolerated with 100% adherence (no drop-outs) until the end of treatment. Patients reported only mild and short lasting adverse effects; eight patients experienced sleepiness, six had headaches during the first sessions and two reported nausea. No serious adverse effects were reported.

### Non-motor activities of daily living (non-motor ADL, MDS-UPDRS part I)

**Figure 1:**
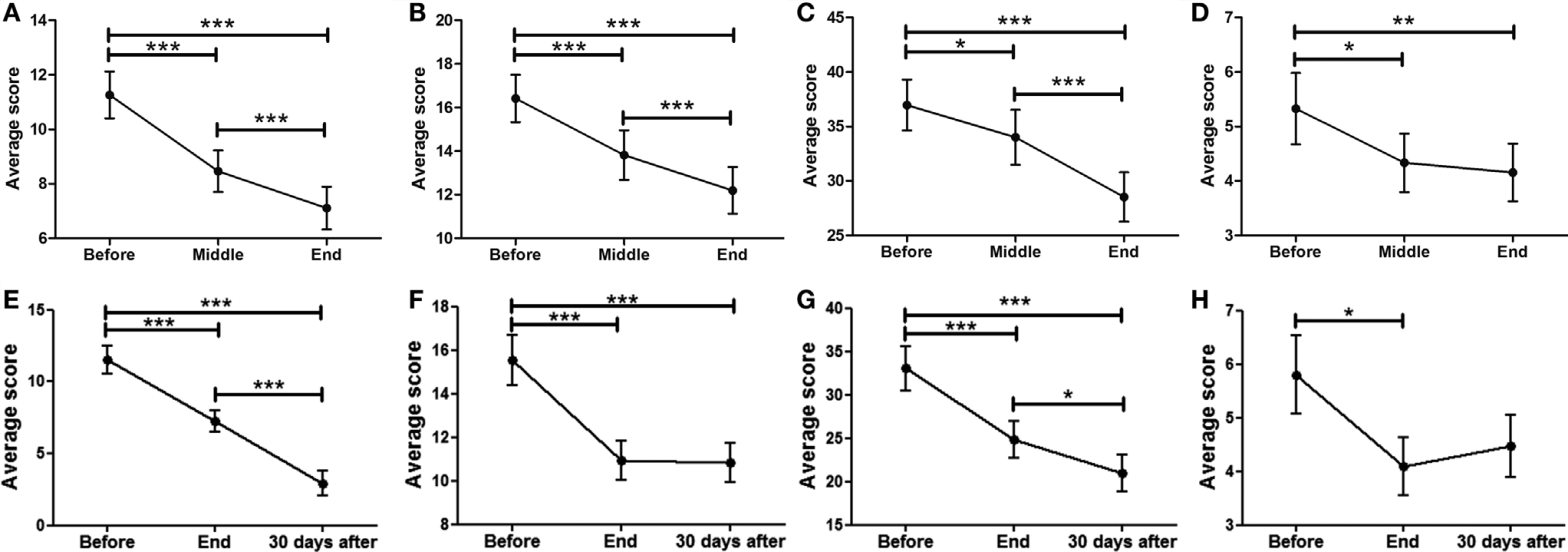
**Effects of dTMS in PD measured using the MDS-UPDRS scale**. dTMS produced significant improvements in non-motor ADL **(A)** and motor ADL **(B)** at Middle and End of treatment. **(C)** dTMS produced a decrease in motor examination (MDS-UPDRS Part III), with a significant reduction at the End of treatment. **(D)** dTMS induced an improvement in motor complications that was significant at the End of treatment. Similar results are shown in a group of patients with 30-day Follow-up, in non-motor ADL **(E)**, motor ADL **(F)**, motor examination **(G)** and motor complications **(H)**, where a significant difference was found in all measures. (*p < 0.05; **p < 0.01; ***p < 0.001).

Data showed a strong, significant correlation between time (*F* = 50.74; *p* < 0.0001) and non-motor ADL (Figure 1A), with a significant point reduction between BEFORE and MID (11.3 ± 0.9–8.5 ± 0.8 points; *p* < 0.001), BEFORE and END (11.3 ± 0.9–7.1 ± 0.8 points; *p* < 0.001), and MID and END (8.5 ± 0.8–7.1 ± 0.8 points; *p* < 0.001). In the case of the patients that attended the 30-day, post-treatment FOLLOW-UP (Figure 1E), they showed a significant decrease (*F* = 8.510; *p* < 0.0001) from BEFORE to END (11.6 ± 1.0–7.3 ± 0.8 points; *p* < 0.001), between BEFORE and FOLLOW-UP (11.6 ± 1.0–2.9 ± 0.9 points; *p* < 0.001), and from END to FOLLOW-UP (7.3 ± 0.8–2.9 ± 0.9 points; *p* < 0.001), suggesting further improvements 30 days post-treatment, beyond the improvements attained by the END of treatment.

### Motor activities of daily living (motor ADL, MDS-UPDRS part II)

A significant relationship between time (*F* = 42.07; *p* < 0.0001) and motor ADL was found (Figure 1B). Significant symptom improvements were found between BEFORE and MID (from 16.4 ± 1.2 to 13.8 ± 1.1 points; *p* < 0.001), between BEFORE and END (reduction from 16.4 ± 1.2 to 12.2 ± 1.1 points; *p* < 0.001), and between MID and END (reduction from 13.8 ± 1.1 to 12.2 ± 1.1 points; *p* < 0.001). For the group of patients that attended the FOLLOW-UP session (Figure 1F), the statistically significant improvement between BEFORE and END (*F* = 18.58; *p* < 0.0001) (reduction from 15.6 ± 1.2 to 10.9 ± 0.9 points; *p* < 0.001) was maintained for over 30 days (BEFORE to FOLLOW-UP; reduction from 15.6 ± 1.2 to 10.8 ± 0.9 points; *p* < 0.001), showing no significant differences between END and FOLLOW-UP.

### Motor examination (MDS-UPDRS part III)

A strong correlation between time (*F* = 24.97; *p* < 0.0001) and motor MDS-UPDRS (Figure 1C) was found. Motor symptoms decreased over time and reached significance at the MID and END time-points compared to BEFORE [37.0 ± 2.3 (BEFORE) to 34.0 ± 2.5 (MID) to 28.5 ± 2.3 (END); *p* < 0.001]. At FOLLOW-UP patients demonstrated a significant decrease (*F* = 11.09; *p* < 0.0001) between BEFORE and END (*p* < 0.001), BEFORE and FOLLOW-UP (*p* < 0.001), and between END and FOLLOW-UP (*p* < 0.05), decreasing from 33.1 ± 2.6 (BEFORE) to 24.8 ± 2.2 (END) and to 21.0 ± 2.2 (FOLLOW-UP). There was a tendency for further improvement after treatment, but no significant differences between END and FOLLOW-UP (Figure 1G). In summary, dTMS treatment correlated with a decrease of 8.3 points in the UPDRS-III at END and 12.1 points at 30 days FOLLOW-UP. Thus, dTMS was a strong predictor for a decrease in PD motor symptoms.

### Motor complications (MDS-UPDRS part IV)

In the present study, patients showed a significant decrease (*F* = 5.35; *p* < 0.01) in motor complications. A significant difference was found between BEFORE and MID as well as between BEFORE and END (Figure 1D), from 5.3 ± 0.7 (BEFORE) to 4.3 ± 0.5 (MID) to 4.2 ± 0.5 (FOLLOW-UP) points (*p* < 0.05; *p* < 0.01). Patients who participated in the follow-up session (Figure 1H) showed similar significant symptom improvements (*F* = 4.99; *p* < 0.01) with a significant difference between BEFORE and END (reduction from 5.8 ± 0.7 to 4.1 ± 0.5 points; *p* < 0.05) and a similar decrease between BEFORE and FOLLOW-UP (reduction from 5.8 ± 0.7 to 4.5 ± 0.6 points), which was not found to be significant. There were no significant differences between END and FOLLOW-UP.

### UP&GO

**Figure 2:**
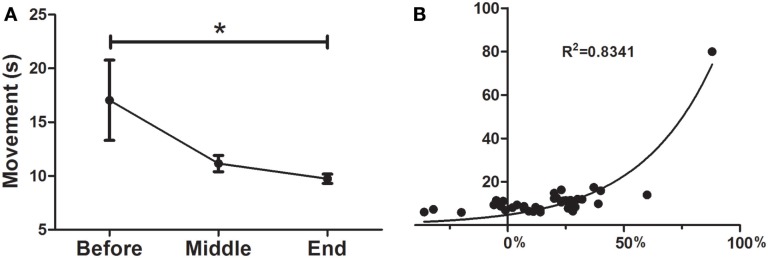
**Effects of dTMS on gait speed (UP&GO)**. **(A)** dTMS produced a significant decrease in the time taken to stand up and walk 3 m, which was significant at the End of treatment, **(B)** The greater the severity in the UP&GO, the greater the improvements after treatment. A correlation was found between initial time in seconds (*y* axis) and the percentage of Deep TMS improvement (initial/final time, *x* axis). Regression shows that the higher the severity, the greater the improvements (*p < 0.05).

As can be seen in Figure 2A the treatment was a significant predictor of improvement in the UP&GO test, as demonstrated by the decrease in the time required to complete the task (*F* = 0.1777; *p* < 0.05), from BEFORE to END (17.0 ± 3.7–9.7 ± 0.4). No differences between BEFORE to MID or from MID to END were found. Interestingly, improvements were correlated with greater severity (*R*^2^ = 0.8341) (Figure 2B), although this result may be explained by a ceiling effect, as patients cannot improve beyond normality, so patients with larger symptom severity will improve more to reach normality than those with less severity.

### TINETTI

**Figure 3:**
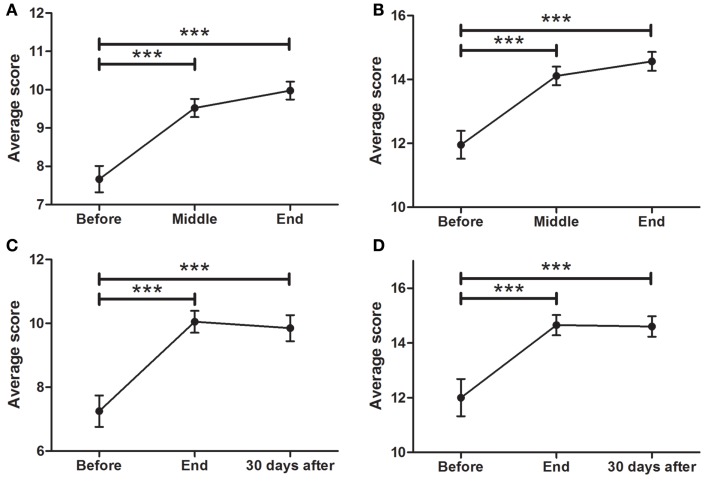
**Effects of dTMS on posture and balance (TINETTI)**. dTMS produced a significant improvement in posture and balance during gait **(A)** and while standing **(B)** at the END of the treatment, which were also significant for balance during gait **(C)** and while standing **(D)** at FOLLOW-UP (30 days after) (***p < 0.001).

Patients showed a significant difference in gait balance throughout treatment (Figure 3A; *F* = 19.67; *p* < 0.0001), increasing from 7.6 ± 0.3 points at BEFORE to 9.5 ± 0.2 at MID (*p* < 0.0001), and to 10.0 ± 0.2 at END (*p* < 0.0001). In addition, there were significant differences in static balance (Figure 3B; *F* = 38.48, *p* < 0.0001), increasing from 12.0 ± 0.3 points at BEFORE, to 14.1 ± 0.3 at MID (*p* < 0.0001), and to 14.5 ± 0.3 at END (*p* < 0.0001).

The improvement in gait balance was maintained at the 30-day follow-up session (Figure 3C; *F* = 13.96; *p* < 0.0001), with significant differences between BEFORE and END (7.3 ± 0.5–10.1 ± 0.3; *p* < 0.0001), and between BEFORE and FOLLOW-UP (7.3 ± 0.5–9.9 ± 0.4). Same was found for static balance (Figure 3D; *F* = 4.066; *p* < 0.0001), in which significant differences between BEFORE and END (12.0 ± 0.7–14.7 ± 0.4) and between BEFORE and FOLLOW-UP (12.0 ± 0.7–14.6 ± 0.4) were found.

### SCOPA-AUT

**Figure 4:**
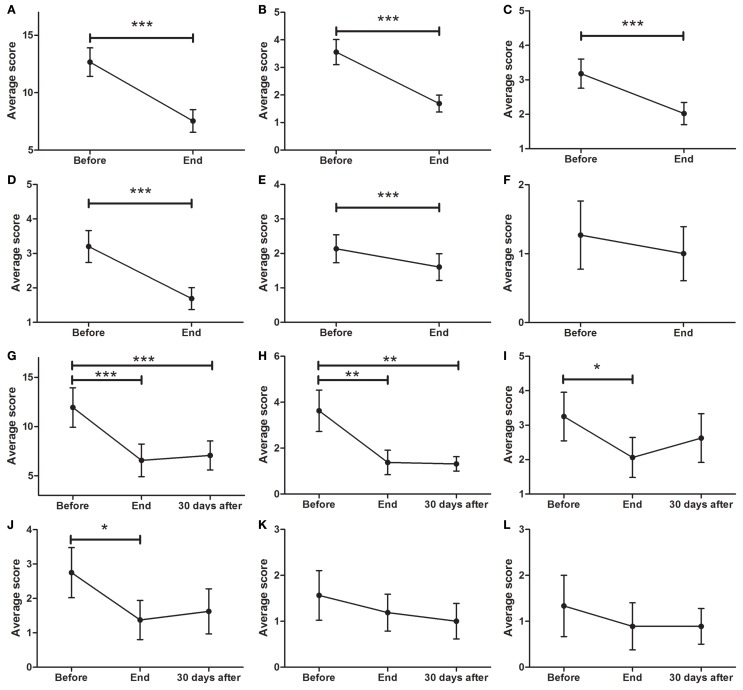
**Effects of dTMS effects on autonomic symptoms as measured by SCOPA**. **(A)** dTMS produced significant improvements in total SCOPA at END. dTMS also induced significant improvements in cardiovascular **(B)**, gastrointestinal **(C)**, urinary **(D)**, and thermoregulation **(E)** from BEFORE to END, but non-significant reductions in **(F)** sexual symptoms. Significant differences were found at the Follow-up period for Total SCOPA **(G)** and cardiovascular symptoms **(H)**, but not for gastrointestinal **(I)**, urinary **(J)**, thermoregulation **(K)** and sexual Symptoms **(L)** (*p < 0.05; **p < 0.01; ***p < 0.001).

As depicted in Figure 4A (*p* < 0.0001), a significant decrease in SCOPA was found between BEFORE (12.7 ± 1.2) and END (7.5 ± 1.0 points, *p* < 0.001). There was also a significant reduction in cardiovascular symptoms (Figure 4B; *p* < 0.0001) from BEFORE (3.6 ± 0.5) to END (1.7 ± 0.3). A significant decrease in gastrointestinal symptoms was found (Figure 4C; *p* < 0.0001) between BEFORE (3.2 ± 0.6) and END (2.0 ± 0.3). Additionally, significant decreases in urinary symptoms were found between the BEFORE and END time points (Figure 4D; from 3.2 ± 0.5 to 1.7 ± 0.3, *p* < 0.001). Thermoregulation also showed significant improvements (Figure 4E; from 2.1 ± 0.4 to 1.6 ± 0.4, *p* < 0.001) but no significant decreases in sexual symptoms were found (Figure 4F).

For patients who attended the follow-up session, the total symptom score (Figure 4G; *p* < 0.0001), showed a significant decrease (*F* = 12.33; *p* < 0.001) from BEFORE to END and from BEFORE to FOLLOW-UP [11.9 ± 2.0 (BEFORE) to 6.5 ± 1.7 (END) to 7.1 ± 1.5 (FOLLOW-UP), *p* < 0.001]. There was a significant reduction (*F* = 8.697; *p* < 0.01) in cardiovascular symptoms (Figure 4H) from BEFORE (3.6 ± 0.9) to END (1.4 ± 0.5) and to FOLLOW-UP (1.3 ± 0.3). Additionally, a significant decrease (*F* = 4.696; *p* < 0.05) in gastrointestinal symptoms (Figure 4I) between BEFORE (3.3 ± 0.7) and END (2.1 ± 0.6) was found. No significant differences were found in gastrointestinal symptoms between BEFORE and FOLLOW-UP. Urinary symptoms showed a significant decrease at END (*F* = 3.575; *p* < 0.05) but not at FOLLOW-UP (Figure 4J; from 2.8 ± 0.7 to 1.4 ± 0.6). There was a non-significant tendency to improve in both thermoregulation (Figure 4K) and sexual symptoms (Figure 4L).

### ON/OFF daily periods

**Figure 5:**
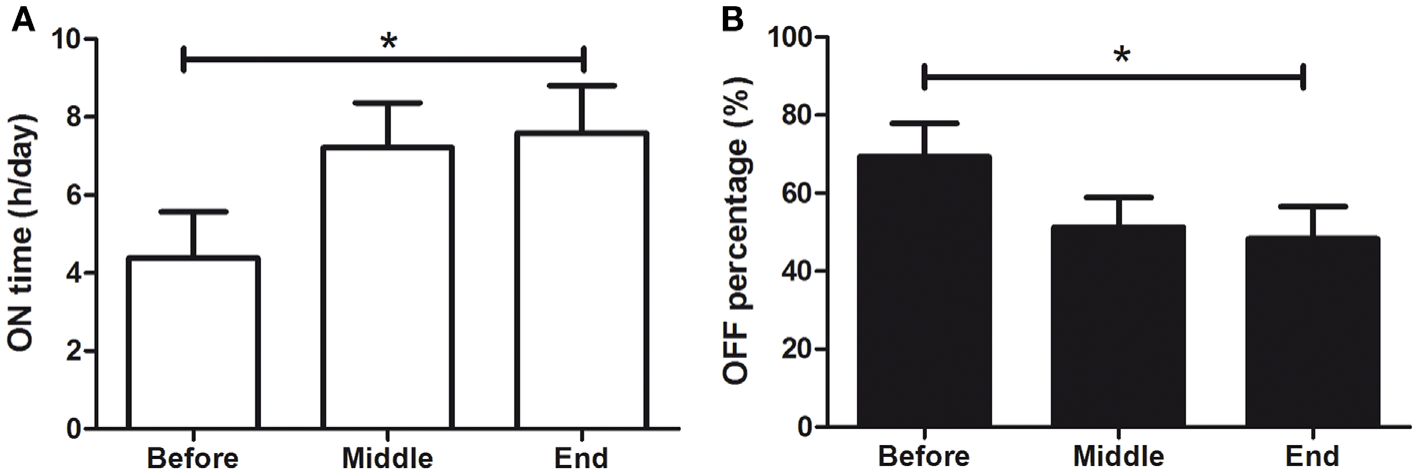
**Effects of dTMS on OFF and ON daily periods**. dTMS produced **(A)** a significant increase of ON time per day and **(B)** a significant decrease in OFF time per hour (*p < 0.05).

Patients were asked to self-assess their daily symptoms and to measure ON & OFF periods every 30 min, from 1 week prior to treatment (BEFORE), until its end (END). Fourteen patients completed the assessment (31% of the patients). There was a significant increase in percentage of ON time, from 32.0 ± 0.8 to 48.0 ± 8.4%/h (see Figure 5A; *F* = 7.619, *p* < 0.001) and a significant decrease in OFF-time from 68.1 ± 8.0 to 52.0 ± 8.0%/h (see Figure 5B; *F* = 18.96, *p* < 0.001). The increase from 32 to 48% of ON time per hour means a 50% increase in ON time each hour. In terms of ON hours per day, patients increased from 4.4 ± 1.2 to 7.6 ± 1.2 h/day, which corresponds to a 73% increase in total ON time per day.

### Hamilton rating scale for depression (HDRS_21_)

**Figure 6:**
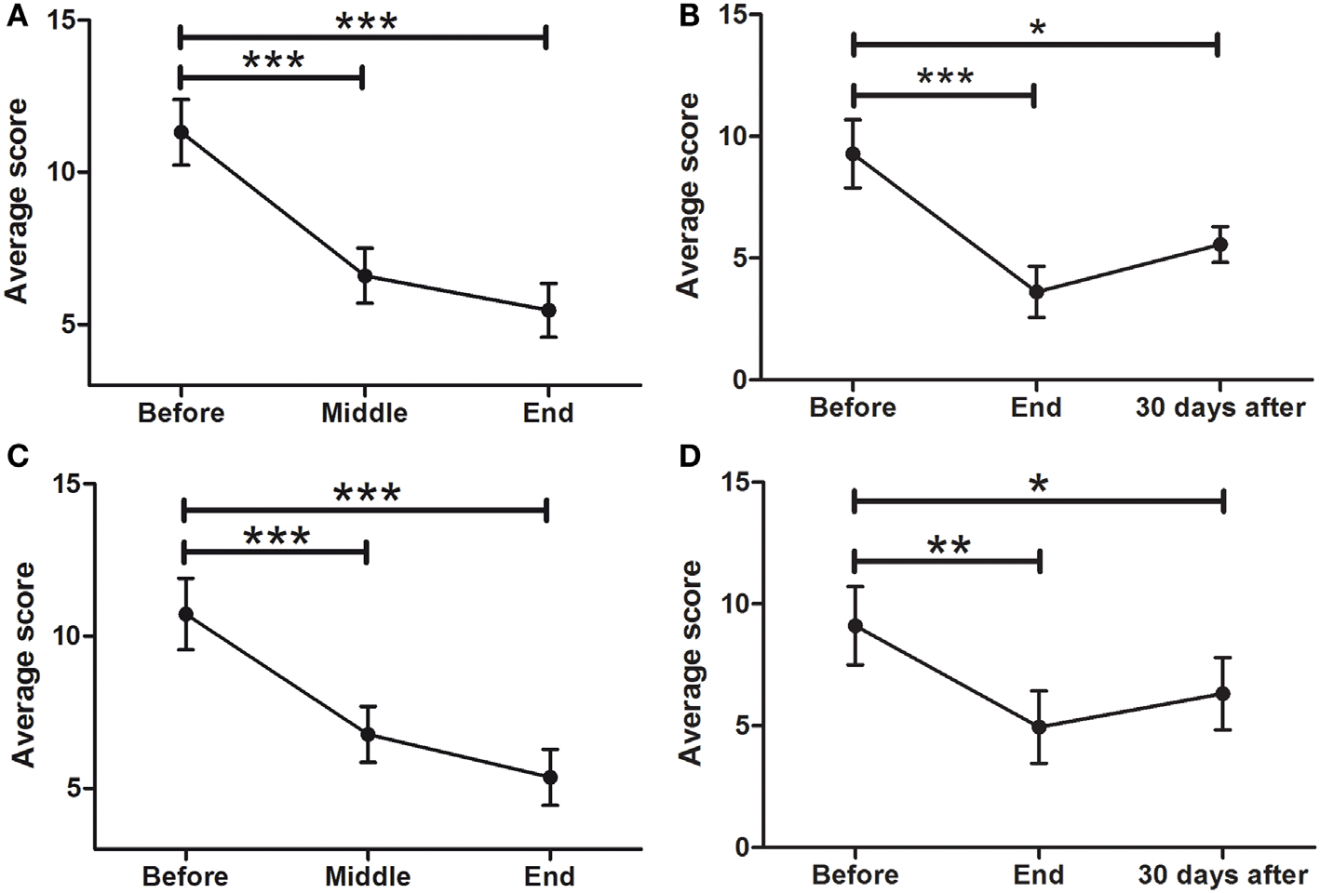
**Effects of dTMS on depressive symptoms as measured by Hamilton (HDRS_21_) and Beck Depression Inventory (BDI)**. dTMS produced a significant improvement in depressive symptoms, leading to remission (<7 points) according to HDRS at the END **(A)** and the FOLLOW-UP **(B)** of the treatment. The same results were found with the BDI at the same points [**(C)** for END and **(D)** for FOLLOW-UP] (*p < 0.05; **p < 0.01; ***p < 0.001).

Of the 45 patients, 29 (64.4%) scored 7 or more points on the *HDRS*_21_. This means that 29 patients in the sample were considered depressed as defined by the physician-applied *HDRS*_21_ (Hamilton) scale. As seen in Figure 6A, there was a significant improvement in overall depressive symptoms of all patients, with a significant effect of time (*F* = 27.64; *p* < 0.0001). There was a significant decrease in depressive symptoms from BEFORE (11.3 ± 1.1 points) to END (5.5 ± 0.9; *p* < 0.0001), improvement that was maintained at FOLLOW-UP (5.6 ± 0.7; *p* < 0.05) (Figure 6B). Analysis of depressed patients only (≥7 points) showed a significant symptom decrease (F014.55; *p* < 0.0001) from BEFORE (12.9 ± 0.9 points) to END (5.6 ± 1.1; *p* < 0.001) in the HDRS scale. At the END time point, 62% of depressed patients experienced symptom remission (<7 points).

### Beck depression inventory

To compare the results of health professional-applied HDRS with a self-applied depression scale, the BDI was used. Consistent with the results found with the HDRS scale, there was also a significant effect of time in depressive symptoms as evaluated by the self-assessed BDI scale (*F* = 25.65; *p* < 0.0001), with a significant decrease from 10.7 ± 1.2 at the BEFORE to 5.4 ± 0.9 at the END point (Figure 6C). This improvement was maintained at the 30 days FOLLOW-UP evaluation [*F* = 9.539: *p* < 0001; from 9.1 ± 1.6 (BEFORE) to 6.3 ± 1.5 (FOLLOW-UP)] (Figure 6D).

### Motor improvements in depressed and non-depressed patients

**Figure 7:**
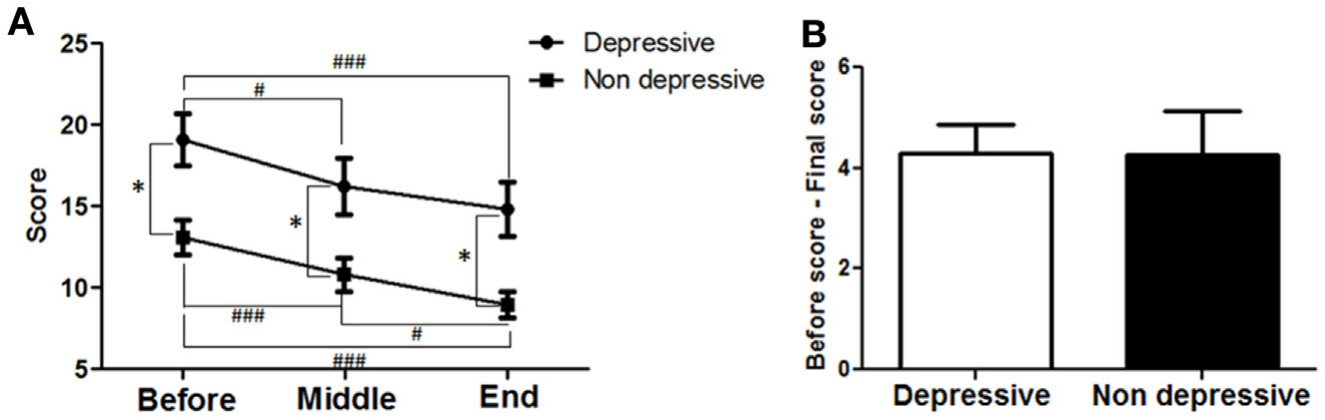
**Effects of dTMS on motor symptoms depending on previous depression as measured by Hamilton (HDRS_21_)**. **(A)** dTMS produced significant and similar improvements in motor symptoms in patients with (≥7 points) or without (<7 points) depression according to HDRS score obtained at the BEFORE session. **(B)** Difference between BEFORE and END in both groups was similar in value and not different statistically (*p < 0.05; **p < 0.01; ***p < 0.001 and ^#^*p* < 0.05; ^##^*p* < 0.01; ^###^*p* < 0.001).

To determine whether antidepressant effects may have contributed to the improvements in motor symptoms, patients’ data were divided into two groups; those that did not have depression (<7 points) and those that had depression (≥ 7 points) as assessed by the HDRS_21_ scale. Twenty-five patients showed depression and 20 were not depressed according to the HDRS_21_ scale. Their data from the motor MDS-UPDRS (Part III) subscale were compared within each group and between groups. A strong correlation between time and motor MDS-UPDRS (Figure 7A) was found for both the depressed (*F* = 28.53; *p* < 0.0001) and the non-depressed groups (*F* = 14.55; *p* < 0.0001). For the depressed group, motor symptoms decreased over time and reached significance at the MID and END time-points compared to BEFORE [19.1 ± 1.6 (BEFORE) to 16.24 ± 1.7 (MID) to 14.8 ± 1.7 (END); *p* < 0.001]. For the non-depressed group, motor symptoms decreased over time and reached significance at the MID and END time-points compared to BEFORE [13.1 ± 1.1 (BEFORE) to 10.8 ± 1.0 (MID) to 9.0 ± 0.8 (END); *p* < 0.001]. Note that depressed patients showed greater symptom severity compared to non-depressed patients, at BEFORE (*p* < 0.001), MIDDLE (*p* < 0.001), and END (*p* < 0.001) time points. The improvements in both groups in response to treatment were almost identical (Figure 7B), showing no significant differences between them (depressed patients, 4.3 ± 0.6 points; non-depressed patients, 4.3 ± 0.9 points). In consequence, dTMS improvements in motor symptoms were significant and similar irrespective of whether patients were depressed or not at the beginning of treatment.

### Correlations between gender, physical activity, severity, and improvements

Multivariate analysis using the Spearman test showed significant correlations between age and initial values of disease severity (MDS-UPDRS motor, non-motor ADL, motor ADL, complications of treatment, UP&GO, and Tinetti). Disease severity according to the H&Y, was correlated with years of disease. Another correlation was found between initial HDRS and both MDS-UPDRS treatment complications and Tinetti. Lastly, a correlation was found between HDRS and ADL at the END time point. The variables age, years of disease or number of sessions treated did not correlate with physical activity. For details see Table 4.

**Table 4:** **Spearman test for multivariate analysis**.

	Age	Gender	Years of disease	Y&H scale	Physical activity	Initial HDRS	Final HDRS	Initial MDS-UPDRS ADL	Final MDS-UPDRS ADL	**Δ** MDS-UPDRS ADL	Initial treatment complications	Sessions
Initial MDS-UPDRS motor	–	–	–	–	–	–	–	***	***	–	***	–
Final MDS-UPDRS motor	–	–	–	–	–	–	*	***	***	–	*	–
Δ MDS-UPDRS motor	–	–	–	–	–	–	–	–	–	–	*	–
Initial UP&GO	–	–	–	–	–	–	–	–	–	–	–	–
Final UP&GO	–	–	–	–	–	–	–	–	–	–	–	–
Δ UP&GO	**	–	–	–	–	–	–	–	–	*	–	–
Initial HDRS	–	–	–	–	–	***	***	–	–	–	–	–
Final HDRS	–	–	–	–	*	***	***	*	**	–	–	–
Initial gastrointestinal SCOPA	–	–	*	–	–	–	–	–	**	–	^–^	–
Δ Gastrointestinal SCOPA	–	–	–	–	–	–	–	–	–	–	*	–
Initial urinary SCOPA	–	–	**	–	–	–	–	–	*	*	–	–
ΔUrinary SCOPA	–	–	**	**	–	–	–	–	–	*	–	–
Initial Cardiovascular SCOPA	–	–	–	–	–	–	–	–	*	**	*	–
Δ Cardiovascular SCOPA	–	–	–	–	–	–	–	–	–	–	–	–
Initial Thermoregulation SCOPA	*	–	–	–	–	*	*	–	–	–	*	–
Δ Thermoregulation SCOPA	–	–	–	–	–	–	–	–	–	–	–	–
Initial sexual SCOPA	–	–	–	–	–	–	–	–	–	–	–	–
ΔSexual SCOPA	–	–	*	–	–	–	–	–	–	–	–	–
Initial Total SCOPA	–	–	–	–	–	–	*	–	**	–	***	–
Δ Total SCOPA	–	–	–	–	*	–	–	–	–	–	–	–
Initial gait tinetti	–	–	–	–	–	–	–	–	–	–	–	–
Δ gait tinetti	–	–	–	–	–	–	*	–	–	–	–	–
Initial static tinetti	–	–	–	–	–	*	–	–	–	*	*	–
Δ static tinetti	–	–	–	–	–	–	–	–	–	–	**	–

Correlations are shown as “*”; no correlation as “–” (*p < 0.05; **p < 0.01; ***p < 0.001).

## Discussion

The present study supports the notion that dTMS could be a safe and effective adjuvant treatment for PD, yet given the lack of a placebo-controlled group, the present study cannot be considered a demonstration of treatment efficacy. However, the efficacy of low frequency stimulation of the motor cortex with rTMS has been demonstrated by several placebo-controlled studies, which were analyzed in a recent meta-analysis (22).

This is the first study to evaluate retrospectively the effects of dTMS in a clinical setting. This is relevant because placebo-controlled studies include only a subgroup of patients with similar symptom severities, whereas in a clinical setting, patients treated show more heterogeneous symptoms, ages, and demographics. The present results, based on a cohort of 45 patients, demonstrate that dTMS using LF stimulation of the M1, and HF stimulation of the PFC is a predictor to a wide range of significant improvements in PD symptoms, including not only motor symptoms, which have been reported previously in placebo-controlled studies for rTMS (22), but also in non-motor, autonomic and depressive symptoms, and significant improvements in activities of daily living (ADL). Although only 32 patients participated in the FOLLOW-UP session, their improvements remained significant 30 days after treatment (see Table 1 for a summary), suggesting that treatment effects lasted at least 30 days post-treatment.

In the recently published meta-analysis that included eight placebo-controlled trials, LF rTMS was reported to be an effective adjuvant for the treatment of motor symptoms in PD, with an average improvement of 5.05 points in the UPDRS part III (22). In the present study, the average decrease in motor symptoms according to the MDS-UPDRS part III subscale was 8.5 points at the END of treatment and 8.6 points 30 days after treatment. The UPDRS is among the most accepted scales available for PD symptom evaluation (29).

Perhaps one of the most important results of the present study is the wide range of symptoms that benefit from dTMS treatment, suggesting that the improvements are not only limited to the motor symptoms included in MDS-UPDRS (III). This suggests that other symptoms could be measured in clinical trials assessing dTMS for PD treatment. For example, the risk of fall, which is common in PD patients and may lead to the need of a walking aid, significantly decreased thereby rendering those aids unnecessary in several patients, affording them the ability, for example, to climb stairs safely.

The present results also suggest that patients benefit from treatment irrespective of disease severity, especially when measuring improvements in the UP&GO scale [which measures gait speed (32)]. The improvements in response to treatment were larger for patients with greater severity, which may be explained by a ceiling effect, as patients with low disease severity have fewer symptoms and thus, less potential for improvement to reach normality, than those suffering from greater disease severity. It is interesting to note that, previous studies using fMRI have shown that rTMS-related improvements in mid-gait freezing are correlated with increased caudate activity and with changes in functional connectivity between PFC and supplementary motor area (9).

The present improvements in autonomic symptoms as measured by SCOPA are consistent with previous studies showing that LF rTMS in M1 or HF in PFC may affect the autonomic system (38), whereas HF in PFC may improve autonomic cardiac dysfunctions linked to depression (39). Our findings further support a possible relationship between the autonomic system and LF rTMS in M1 or HF in PFC.

In terms of depressive symptoms, the self-administered BDI scale was used to contrast patients’ self-perception of mood and depressive symptoms, with clinician-applied HDRS_21_. Both scales revealed significant improvements, reaching remission in 62% of depressed patients (18 out of 29 depressed patients). This antidepressant effect may be the result of motor symptom improvement, as the multivariate analysis showed a correlation between HDRS and ADL at END, suggesting that greater improvements in ADL may lead to greater improvements in depression. However, a previous clinical trial evaluated the benefit of rTMS in PD patients with depressive symptoms, with HF stimulation targeting the left PFC (40) to obtain antidepressant effects. Thus, it is also possible that in the present study the bilateral HF stimulation of PFC contributed to the treatment antidepressant effects.

This raises the question of whether the improvements in motor symptoms obtained here after dTMS can be attributed to antidepressant effects. To answer this question, we compared the improvement of patients that initiated the treatment with and without depression. Our results show that although depressed patients show in average greater motor symptom severity, their improvements are similar to those obtained in patients without depression, at least for the motor UPDRS. This suggests that motor symptom improvements after dTMS are significant and similar irrespective of depressive symptoms. This does not rule out a contribution of antidepressant effects, but demonstrates that motor symptom improvements after dTMS cannot be attributed to antidepressant effects, as patients without depression have similar symptom improvements as those with depression.

Two key factors to be considered in PD studies are time of day and the patients’ emotional state when symptoms are being measured. In this study, measurements could not be performed during OFF periods as patients ranged from those experiencing <10% of the day OFF, to those experiencing full day OFF. Patients with 100% OFF response to Levodopa were included in the present study only if diagnosed with PD and had been responsive to the drug earlier in their disease. Measurements were taken at the same time of day in all patients, during an ON period, unless the patient experienced no ON periods, in which case measurements were taken at the time most comfortable to the patient. To ensure that measurements did reflect the patient’s motor symptoms throughout the day, they were given a self-applied scale for ON&OFF periods with questions to be answered every 30 min. Adherence to scale completion reached 30%, and resulted in two major findings. First, there was a significant and large increase in daily ON time, incrementing from 4.4 ± 1.2 to 7.6 ± 1.2 h/day, a 42% increase. Second, 5 patients began treatment with 100% OFF time (without ON time) and by the end of treatment, 3 of those patients had gained ON periods, increasing in average from 0 to 6.3 h/day. This implies that there was a significant improvement in the amount of ON hours per day independently of the time of measurement, suggesting that dTMS may potentiate the effect of Levodopa. There were no changes in pharmacotherapy before or during treatment. Although dyskinesia decreased in average during treatment, four patients showed increased dyskinesia after treatment. We were able to control this increase by decreasing levodopa dosage post-treatment. In consequence, it may be proposed that dTMS treatment potentiates the effects of levodopa, which in some cases may lead to a decrease in the necessary levodopa dose and may control the medication’s complications. No matter how suggestive these results may seem, a placebo-controlled, double blind study should be performed, specially designed to determine the efficacy of treatment in non-motor symptoms and to assess the possibility that treatment potentiates levodopa effects.

The present study has several limitations; it is a retrospective evaluation of clinical efficacy, which means it is a compilation of 45 clinical cases on the effects of dTMS for the treatment of PD symptoms. Thus, there was no control group to measure the placebo effect and therefore it was not “blinded.” This lack of placebo control is important. Previous studies have shown that significant dopamine release may occur when the declared probability of receiving active treatment was above 75%, concluding that the strength of belief of improvement can directly modulate dopamine release in patients with PD (41). Placebo responses in PD have been reported with a magnitude between 9 and 59% of that of the active drug group (42), or 7–10% of symptom relief (43), as well as subjective and short lasting, not measured consistently by currently used scales (44). Although the present study does not have a placebo control group, the treatment with LF rTMS of motor cortex has been shown to be effective against placebo in a series of randomized placebo-controlled clinical trials reviewed by Zhu and colleagues (22). The above placebo-controlled studies were designed to assess improvements in motor symptoms using UPDRS and were not designed to test non-motor symptoms, which according to the present study, may also show improvements in response to treatment.

The SCOPA, MDS-UPDRS, and TINETTI scales used here are widely accepted and used in previous studies. The multivariate analysis showed correlations between initial values of the scales used (MDS-UPDRS, SCOPA, TINETTI) *and* disease severity *and* years of disease, as well as a correlation between ADL, SCOPA, and depression. All those correlations indirectly validate the scales used, as such correlations are expected; for example, ADL should correlate with depression, and motor exploration should correlate with disease severity. Expected correlations were also found between disease severity and years with disease, in addition to a correlation between ADL, SCOPA, and depression.

A few interesting results were found using the Spearman test. First, there was a correlation between physical activity and both the score in HDRS at the END and the difference (improvement) in the total SCOPA score. By contrast, gender or physical activity did not correlate neither with disease severity or treatment results, whereas patients who exercise may improve more in autonomic symptoms and end treatment with greater improvements in depression.

An issue that cannot be readily assessed in the present study is the duration of the improvements. The present results suggest that benefits from treatment last for at least 30 days. However, a placebo-controlled study is required to assess the duration of dTMS effects in PD.

In conclusion, dTMS is a strong predictor of improvements in PD symptoms. In our cohort of 45 patients, dTMS treatment induced significant improvements in motor and non-motor symptoms, ADL, gait, posture, balance, risk of fall, gait speed, autonomic and depressive symptoms, as well as a 42% increase in ON hours per day. Improvements were independent of subjacent depression. These findings suggest that dTMS may be an effective add-on for the treatment of PD. A placebo-controlled trial analyzing the effects of dTMS on these symptoms need follow this study to demonstrate the efficacy of dTMS in PD and possible synergic effects with concomitant dopaminergic treatment.

## Conflict of Interest Statement

FT, EV, and PP work at NeuroMagnetics SA. RR and JS are medical and scientific consultants for NeuroMagnetics SA and have financial interests in NeuroMagnetics SA, the representative of Brainsway Inc. in Chile. AZ serves as a research consultant and has financial interest in the Brainsway Inc. RM-A and SL have no conflicts of interest.
